# Exploring Resiliency and Parentification in Polish Adolescents

**DOI:** 10.3390/ijerph182111454

**Published:** 2021-10-30

**Authors:** Piotr Połomski, Aleksandra Peplińska, Aleksandra Lewandowska-Walter, Judyta Borchet

**Affiliations:** Institute of Psychology, Faculty of Social Sciences, University of Gdańsk, 80-309 Gdańsk, Poland; piotr.polomski@ug.edu.pl (P.P.); aleksandra.peplinska@ug.edu.pl (A.P.); aleksandra.lewandowska-walter@ug.edu.pl (A.L.-W.)

**Keywords:** resiliency, current parentification, adolescence, gender, Poland

## Abstract

Parentification is a form of distorted division of roles and responsibilities in the family where the roles of parent and child are reversed. A situation that goes beyond the child’s capabilities and exhausts resources usually yields numerous negative consequences. Nevertheless, in some circumstances, parentification may be beneficial by shaping resiliency. The main aim of the study was to examine the relations between parentification characteristics and resiliency. There were 208 adolescents (*M_age_* = 14.55; *SD_age_* = 1.00) who participated in the study. Resiliency was evaluated using the Polish Scale for Children and Adolescents SPP-18. Parentification level was measured with the polish Parentification Questionnaire for Youth. The analyses revealed significant relations between parentification and resiliency dimensions. The relations were different based on the participant’s gender. The obtained results underline the role of resiliency in shaping the perception of family role dysfunctions such as parentification.

## 1. Introduction

### 1.1. Resiliency

The term resilience was introduced in the 1950s by Jeanne and Jack Block [1,2], contributing to the initiation of numerous studies of this phenomenon that continue up until today. The multiplicity of analyses and concepts that arose around broadly understood psychological resilience stems partly from a lack of consensus among scholars regarding the nature of this concept. The meaning of resilience evolved over time, as it was studied in more depth [3]. Moreover, in the field of Polish psychology, there is no consensus regarding the term’s translation. Resilience has been translated as “sprężystość” [elasticity], “sprężystość psychiczna” [psychological elasticity], “odporność na zranienie” [resistance to wounding], “prężność” [resilience], and “prężność osobowa” [personal resiliency] [4].

Currently, two approaches appear to dominate publications on the subject—looking at resilience as a process and resiliency/resilience as a personal quality of an individual. The first approach considers resilience as a process of dynamic, positive adaptation of an individual in response to obstacles—authors advocating this perspective claim that activating resilience requires experiencing direct danger or a traumatic event. The process of resilience activation allows an individual to deal with a difficult situation by mobilizing appropriate competencies and capabilities [5].

The second approach considers resiliency as a personal quality of an individual or a relatively durable psychological resource and is referred to as psychological resiliency and personal resiliency. It plays an important part in the process of coping with both traumatic events and everyday life. This approach to resiliency originates from Block’s [2] theory of ego-resiliency that defines it as a “relatively enduring, structural aspect of personality” that determines the process of elastic adaptation of an individual to changing life requirements. According to the author [6], resiliency enables effective, flexible, and creative adaptation to changing circumstances. In this sense, resiliency allows an individual to introduce effective changes in their personality and behavior, as well as to retain the sense of control and distance towards one’s emotions. Ego-resiliency is the ability to restore good functioning of an individual, participate in social life, and cope with difficult situations.

Ogińska-Bulik and Juczyński [1], whose approach serves as the frame of the present article, treat resiliency as a self-regulating mechanism that consists of three elements—cognitive, emotional, and behavioral. The cognitive components encompass beliefs and expectations of an individual (i.e., regarding one’s competencies), and translate to, for example, perceiving reality as a set of challenges. According to Ogińska-Bulik and Juczyński [1], emotional components of resiliency are connected to experiencing positive affect and emotional stability. Behavioral components of resiliency manifest in searching for new experiences and using effective coping strategies when dealing with problems and difficulties. Understood in this way, resiliency is conducive to perseverance and adaptability to life expectations, as well as to mobilizing an individual to take countermeasures and to reduce susceptibility to experiencing negative emotions and failure. In this context, resiliency may be connected to a positive approach to life, emotional stability, and perceiving difficulties as opportunities enabling one to gain new experiences [7].

Resiliency plays an important part in the functioning of children and adolescents. While most studies in the area have considered resilience as a process [1], some of them look at resiliency in children and adolescents as a quality of an individual, their personal resource. According to the latter analyses, high resiliency is connected to higher reasonableness of actions undertaken by the children, a positive attitude to life, a higher level of autonomy and trust towards oneself, as well as more efficiency in performing everyday tasks [8]. Highly resilient children exhibit more interpersonal skills, which facilitate forming nurturing relationships with others [8,9]. Moreover, students with higher resiliency levels are stronger focused and more attentive during lessons as well as more willing to help and cooperate than their less resilient peers [10]. Other studies of adolescents have shown that ego-brittle individuals (i.e., meaning the opposite of a resilient one) have shown more depressive symptoms and susceptibility towards problematic behavior, including the usage of drugs [11]. Studies conducted in Japan [12] have shown that resiliency in teenagers is positively correlated with self-esteem and mental well-being [13]. Moreover, high resiliency prevents teenagers from risky and health-threatening behavior [14].

### 1.2. Parentification

Parentification is a common, complex, but increasingly better-understood phenomenon that affects many children and adolescents and consists in reversing the roles in a parent-child dyad [15,16,17]. The reasons consist of incorrectly serving the parental role by an adult [18] that has been initially associated with a number of circumstances as risk factors for the occurrence of a situation in which children perform the roles and tasks of adult members: a chronic disease of a family member (parent or siblings; [19,20,21]), a parent being addicted to a psychoactive substance [22,23,24,25], divorce [26,27,28], and family migration [29,30].

The course of parentification and the consequences of its various types depend on several factors. Those can be, for example, the child’s age at which he or she was parentified and the duration of the situation in which the child performed the duties of a parent, as well as the child’s gender and individual predispositions. The earlier a child experienced a disruption in the family’s hierarchy and the longer it lasted, and also the more often the child performed age-inappropriate tasks inadequate in terms of its capabilities, the more severe were the consequences of parentification [15,20,31]. The emergence of obligations towards the family at an older age, short-lasting burdens, and performing tasks coherent with cultural norms might have worked in favor of perceiving the situation as less stressful and more just by the child [17,20,32].

The consequences of reversing family roles in childhood and adolescence may be impactful for the individual’s future life and may consist in postponing the undertaking of tasks typical for adulthood [33]. Due to the dynamics of changes, adolescence seems to be particularly sensitive to the emergence of additional risk factors, the constellation of which may result in functional disorders. At the same time, an adolescent can, to a greater extent than a child, seek and take advantage of external sources of support in the form of peers, teachers, and other adults from the family of origin. Relationships with these people may become a protective factor minimizing the consequences of parentification [34,35]. As it results from research devoted to the significance of relations with siblings in the situation of parentification, the closeness with a brother or sister may also constitute an important protective factor during this period of development [36].

Parentification does not have to affect all children in the family system. A child could have been “chosen” for the role by the parent, for example, due to its individual predispositions and gender, as well as the similarity to the parent in terms of these two factors [37,38]. The child that is most sensitive to the expectation of help from a parent is often the firstborn [39], and the oldest daughters are also included in a special risk group [17,40]. It is possible to notice tendencies to more often choose girls as caretakers due to the socialization process in which they are being prepared to help others and provide care for their loved ones [41]. Boys are also being burdened with parentification and experience its negative effects such as lower readiness to verbalize the fact of serving the role of a caregiver due to perceiving it as non-masculine [20,42,43]). Burton [44] also notices that boys more often caregive in an instrumental way, while girls are entrusted with functions concerning emotional caring for the family.

Thus, parentification may be related to gender as well as mechanisms of coping with the parentification may depend on the type of activity (dimension of parentification—emotional or instrumental) performed by the adolescents in favor of the family [45], as well as the accordance of these tasks with the stereotypically perceived gender roles [46].

The negative consequences of parentification, such as high levels of anxiety, depression, personality disorders, or psychoactive substances overuse, are extensively documented in the literature [18,22,47,48]. Research concerning constructive parentification, in which the child’s difficult situation is understood as one in which it has a chance to reveal and develop its potential, is of less interest. Existing studies indicate such benefits as increased self-esteem, empathy, and altruism [15,36,45,49] as well as overall life satisfaction [50].

### 1.3. Relations between Parentification and Resiliency/Resilience

Studies on resilience consider it as a complex factor that, depending on the level of parentification, can be both the predictor of parentification outcomes [51] or the outcome itself [49,52,53]. For example, parentification was generally related to resilience and predicted a mild level of posttraumatic growth [54]. Moreover, parentification predicted adaptive coping in children of HIV-positive parents after six years [51]. Woolgar and Murray [55] notice that in the families where parentification emerges, not always both of the parents present psychopathological symptoms. Thus, the child’s perception of the parent that has trouble performing the parental role may not influence the child’s perception of the other parent, and this parent may enable the resilience process [56]. If the child’s needs are met, the child receives support, and the demands toward the child are age-appropriate, despite experiencing adversities within the family, the child may respond resiliently [54,55].

As Walker and Lee [56] noticed in their work on the parentified children of a parent with alcohol abuse, perceiving them through the prism of the adversities they face and deficit point of view is reductionist. Those children still have opportunities to learn adaptive capability and develop nurturing and supportive relations with other people (e.g., siblings or the parent without substance dependency), and their parentification may be a sign of relational resiliency (i.e., parentification may benefit the family system). Moreover, teachers and mentors may shape resilience in students who experience parentification [57].

However, the optimism about the positive outcome of parentification should be cautious, as it is highly contextual. For example, in a study of parentification among young adults, Boumans and Dorant [58] revealed that young carers did not present higher resilience than the non-carers. As Walker and Lee said

“*Therapists need to be sensitive to the constructive functions that parentification can serve when COAs (COAs—children of alcoholics) are not overwhelmed with that role. Although some degree of parentification may indicate a family’s relational resilience, therapists also should not be naive. Role reversals can be exploitative if the child’s role is not supported, periodically relieved, or reciprocated by parental figures*”.

[56]

The aim of the current study was to explore the relations between current parentification and resiliency in a sample of Polish adolescents. Thus, two research questions were put forward: (1) Are parentification and resiliency different in girls and boys?; and (2) are parentification and resiliency related? To answer the research questions, we have performed sequences of *t*-tests and *r*-Pearson correlation analysis.

## 2. Materials and Methods

### 2.1. Procedure

Prior to the study, ethical approval from the Ethics Committee for Research Projects at the Institute of Psychology of the University of Gdansk was received (#06/2017, dated 10 December 2017) as well as the parental and participant’s consent. The study was conducted during the 2017/2018 school year. The students were asked to fill in a set of questionnaires during one of their classes. The procedure lasted from 25 to 30 min. After the students finished completing the questionnaires, they were thanked for their participation in the study. Afterwards, there was time for them if they had any questions.

### 2.2. Participants

The study was performed in two public schools in Gdańsk, Poland. Originally, there were *N* = 208 adolescents who took part in the study. The sample inclusion criterion was having at least one sibling, and this criterion aimed to ensure sample homogeneity (i.e., everyone has at least one sibling) and enable analyses of parentification focused on siblings. However, not all participants from the original sample met the sample inclusion criterion (i.e., having at least one sibling).

Therefore, *n* = 44 participants (i.e., adolescents who were the only children in their families) were removed from the sample. The final sample consisted of *N* = 164 adolescents, and all of them met the inclusion criterion, which was having at least one sibling.

The participants were aged 13 to 16 years old, with a median of 15 (*M_age_* = 14.55; *SD_age_* = 1.00). In the target sample, girls constituted 61.6% (*n* = 101) of the sample, boys 38.4% (*n* = 63). The age difference between girls (*M_age_* = 14.49; *SD_age_* = 0.97) and boys (*M_age_* = 14.65; *SD_age_* = 1.05) was not statistically significant (*t*(162) = −1.033, *p* = 0.303). All of the participants had siblings and 51.8% were the firstborns (31.1% were the youngest sibling, 17.1% were the ‘middle’ sibling). Most of the study participants lived with both their parents (i.e., 81.1%). On a scale from 1 to 10, the mean subjective family socioeconomic status assessed by the adolescents was *M* = 6.53; *SD* = 1.29.

### 2.3. Measures

The study employed two Polish-language questionnaires: SPP-18—a Polish scale to measure resiliency in children and adolescents [1] and PQY—Parentification Questionnaire for Youth [59]. The participants were also asked about their demographic information such as age, gender (girl/boy), sibling status (yes/no), and family type (living with both parents/living with mother/living with father/living with mother and her partner/living with father and his partner).

***Resiliency.*** SPP-18 [1] is a measure developed for children and adolescents (aged from 12 to 19) that allows for resiliency measurement. The scale consists of 18 items, rating on a 5-point Likert-type scale (from 0 meaning ‘*totally disagree*’ to 4 meaning ‘*totally agree*’). The scale produces 4 subscales: (1) personal competencies and negative affect tolerance, (2) sense of humour and openness to new experiences, (3) persistence and determination in action, and (4) optimistic attitude and energy. The scale also enables to calculate a total score (general gesiliency). Scores are calculated as the mean of the ratings for the subscale items. Reliability for the total score (α = 0.91) and subscale scores in this study were sound (i.e., Cronbach’s α ranged from 0.78 to 0.83).

***Parentification.*** Parentification Questionnaire for Youth (PQY; [59]) is a questionnaire allowing for the measurement of current parentification in adolescents (aged from 12 to 18). The questionnaire consists of 26 items rated on a 5-point Likert-type scale (from 1 meaning ‘*never true*’ to 5 meaning ‘*always true*’). The scale builds up 6 subscales–4 general subscales (emotional parentification toward parents, instrumental parentification toward parents, sense of injustice, and satisfaction with the role) and 2 subscales for adolescents who have siblings (i.e., instrumental parentification toward siblings and emotional parentification toward siblings). Scores are calculated as the mean of the ratings for the subscale items. The questionnaire does not produce a total score [59]. The reliability of the subscale scores was sound in this study (i.e., Cronbach’s α ranged from 0.72 to 0.80).

### 2.4. Planned Data Analysis

Prior to the analysis, descriptive results, r-Pearson correlations between studied variables, and reliability of the measures (Cronbach’s α) were assessed. Those results are presented in Table 1. All measures were reliable that allowed for further exploration of the data.

As the current study is the first study that explores the relationship between parentification and resiliency in Polish adolescents, we lacked the basis to build complex analytical models, and we decided to perform a simple exploration. In order to answer the first research question (Are parentification and resiliency different in girls and boys?), a sequence of *t*-tests was performed. For the purpose of answering the second research question (Are parentification and resiliency related?), we employed the *r*-Pearson correlation analysis. All analyses were performed using Statistical Package for Social Science (SPSS) 26.

## 3. Results

### 3.1. Step One: Gender Differences in Resiliency and Parentification

The first step of the analysis was to compare the resiliency and parentification subscale scores between girls and boys. A sequence of the independent samples *t*-tests was performed. The results are presented in Table 2.

Within the resiliency dimensions, boys scored higher than girls on 3 out of 5 SPP-18 scales [1], which were: personal competencies and negative affect tolerance (*t* = 3.34; *p* < 0.01), persistence and determination in action (*t* = −2.20; *p* < 0.05) and general resiliency (*t* = −2.97; *p* < 0.05). Therefore, in the studied sample, boys presented higher resiliency in general as well as more features favoring coping with various hardships such as the abilitiy to cope and tolerate negative experiences, as well as persistence and determination while fighting obstacles.

Within the parentification dimensions [59], the analyses revealed 4 statisctically significant differences. Girls presented a higher level of emotional parentitfication focused on parents (*t* = 2.01; *p* < 0.05) as well as instrumental parentification toward siblings (*t* = 3.17; *p* < 0.01), whereas boys reported a higher level of instrumental parentification focused on parents (*t* = −2.20; *p* < 0.05) and higher satisfaction with their family role (*t* = −2.07; *p* < 0.05) than girls did. Summing up, gender differentiated the levels of both resiliency and parentification dimensions.

### 3.2. Step Two: Zero-Order Correlation Analysis

Based on the results of the first step of the analysis, we decided to conduct the analyses of the relationships between resiliency and parentification dimensions separately for the girls and for the boys. We performed r-Pearson correlation analyses. The results are presented in Table 3 and Table 4.

For the girls, the correlation matrix revealed many statistically significant moderate associations between SPP-18 and PQY subscales (Table 3). Firstly, persistence and determination in action was positively associated with emotional parentification toward parents (*r* = 0.27, *p* < 0.01), satisfaction with the role (*r* = 0.32, *p* < 0.01), instrumental parentification toward siblings (*r* = 0.31, *p* < 0.01), and emotional parentification toward siblings (*r* = 0.21, *p* < 0.05). At the same time, persistence and determination in action was negatively related to sense of injustice (*r* = −0.23, *p* < 0.05). Optimistic attitude and energy was negatively related to the sense of injustice (*r* = 0.39, *p* < 0.01) and positively related to satisfaction with the role (*r* = 0.37, *p* < 0.01). Girls’ general resiliency was positively related to satisfaction with their family role (*r* = 0.25, *p* < 0.05), instrumental parentification toward siblings (*r* = 0.20, *p* < 0.05), and emotional parentification toward siblings (*r* = 0.21, *p* < 0.05).

For the boys, the correlation matrix revealed statistically significant moderate associations between SPP-18 and PQY subscales (Table 4). However, some of these associations were different from those observed in girls.

The three associations between the variables that were present in boys (i.e., in boys only) were: the relation between personal competencies and negative affect tolerance and satisfaction with the family role (*r* = 0.28, *p* < 0.05); the relation between optimistic attitude and energy and emotional parentification toward siblings (*r* = 0.36, *p* < 0.01); and the relation between general resiliency and instrumental parentification toward parents (*r* = 0.26, *p* < 0.05).

The associations that were present in both groups (i.e., in girls and boys) included: positive relations between persistence and determination in action and instrumental parentification toward parents, emotional parentification toward parents, satisfaction with the role, instrumental parentification toward siblings, and emotional parentification toward siblings (*r* ranging from 0.32 to 0.38); relations between an optimistic attitude and energy and instrumental parentification toward parents (*r* = 0.31, *p* < 0.05) and emotional parentification toward parents (*r* = 0.33, *p* < 0.01); and relations between general resiliency and satisfaction with the role (*r* = 0.25, *p* < 0.05) and emotional parentification toward siblings (*r* = 0.45, *p* < 0.01).

Moreover, some relations between resiliency and parentification dimensions were not present in boys but were present in girls (i.e., in girls only). Those included the relation between persistence and determination in action and sense of injustice; the relations between optimistic attitude and energy and sense of injustice as well as satisfaction with the role; and the relations between general resiliency and sense of injustice as well as instrumental parentification toward siblings.

## 4. Discussion

The study had a novel goal to explore the relation between resiliency and parentification in the sample of Polish adolescents for the first time. It is important to explore the parentification process in the group of Polish adolescents because so far, most of the Polish studies on parentification concerned adults or emerging adults, and those studies were retrospective [23,24,32,36]. Moreover, a former nationwide study conducted among Polish adolescents aged 11–17 showed that 72% of the respondents experienced at least one form of abuse or neglect [60]. These events could have been connected to various family environments and structures that potentially may have served as a source of reversing roles in the family (e.g., substance abuse, divorce, parental conflict, or working late hours). Moreover, in a study by Kobylarczyk and Ogińska-Bulik, adolescents indicated that the most difficult event that they experienced was the loss of a loved one, followed by their own chronic or acute illness or that of a family member, divorce of their parents, and severe financial difficulties [61]. Some of these events represent risk factors of parentification because those are situations when a child has to perform the role of a parent to make up for a parent’s mental or physical absenteeism [62].

The experience of difficult life events, in addition to many negative consequences, may also entail the occurrence of positive changes, expressed in the form of an increase in the level of resilience or even posttraumatic growth [54]. As Hooper [34] points out, in the last thirty years of research on parentification, the focus was primarily on its adverse effects. There is still little research that will allow us to understand the mechanism of the development of potentially positive effects, which is another indication of the concept of the discussed research issues, also in the group of adolescents.

The results of the current study indicated that resiliency dimensions were related to parentification dimensions, and some of these relations might be gender-specific (e.g., shaped by the cultural norms related to the girls’ and boys’ upbringing and expectations toward them). The obtained findings are consistent with the reports of predecessors indicating the positive relationship between parentification and resilience/-y [49,52,53,54,57,63]. This finding supports the assertion of Kuperminc et al. [29], who described the influence of parentification as “competence at a cost”. In Poland, because of strong cultural norms which place a high value on the family unit, children may be motivated to support their family [64]. Individuals who are taking the role of the family carer may be positively reinforced by the family and their environment because of the cultural influences. Thus, it is important to take the cultural context under consideration to better understand the outcomes of parentification [65].

Resilience refers to the ability to reduce the negative effects of hardship and to bring about positive change in negative circumstances. The results of the current study show that the distinguishing positive correlate of parentification in the group of Polish adolescents was perseverance and determination (as a dimension of resilience). Although there are no studies that would directly indicate the relationship between the two discussed variables, there are those that show that parentification correlates with academic motivation [57], academic achievement [57,66], effective task planning and goal-oriented coping strategies [54], as well as with self-efficacy [67], which are indirectly related to persistence and determination. Additionally, long-term observations of Meichenbaum [68] show that overcoming the difficulties that arose in a child’s life may foster his or her resistance. At the same time, it is worth paying attention to the risk of crossing the line of perseverance and determination towards overloading, as Jurkovic [15] and Winton [69] pointed out that children and adolescents experiencing parentification may show tendencies towards perfectionism, workaholism, and overachieving.

## 5. Implications

Regardless of the fact that in the current sample parentification was correlated to resiliency, the results should be interpreted with caution. Due to the dynamics of the individual’s development, in the case of research involving adolescents, it is also worth considering the potentially negative correlates of parentification and the consequences of their current parentification experience in the future, which may also appear after the period of latency.

Resilience may manifest itself in the face of a difficult event or family situation that the teenager is currently experiencing. However, as DiCaccavo [48] points out, individuals with the experience of parentification come to therapy due to mental exhaustion and breakdown caused by the feeling of being unable to continue helping their loved ones. According to more recent studies [58], youth can cope with a difficult situation with the use of less effective avoidance strategies in the long term, which may result in an accumulation of tensions and unworked problems. Therefore, it is important to provide care and support to adolescents who may be at risk of parentification in order to monitor the dynamics of their development and their family situation. Reports indicating the importance of perceiving the situation of parentification and giving it meaning [66,70] may suggest that participation in cognitive-behavioral therapy may be beneficial for people who experienced parentification in childhood and adolescence.

## 6. Limitations and Future Directions

The current study was not free of limitations. Firstly, it was a cross-sectional study, so we can not infer a causal relation between the variables. On the other hand, the study was highly exploratory as it was the first attempt to assess the relations between resiliency and parentification among Polish adolescents, EBSCOhost search (15 May 2021) for the phrase ‘*Poland parentification resilience adolescent*’ or ‘*Poland parentification resiliency adolescent*‘ produced 0 results, and search for the phrase ‘*Polska parentyfikacja prężność*’ produced 1 result but it was a study on adults [24]. Given the lack of Polish empirical reports on the subject, the authors took a cautious and simple analytical approach.

Another limitation of the study is that it did not control for factors shaping the parentification experience, such as, for example, the age at which the child was parentified and the duration of parentification, as those factors are being known to be important in shaping the severity of parentification consequences [15,20,31].

Future studies on positive outcomes of parentification are highly needed [54], especially those which involve understudied populations such as Polish adolescents. Future works on the subject could explore in-depth the mechanism that differentiates the relations between resiliency and parentification in Polish adolescent girls and boys. The authors suggest that the adolescent’s internalization of the gender stereotypes may be involved within this process.

In subsequent studies, it is also worth looking for intermediary variables in the relationship between parentification and resilience because previous studies have shown that negative effects of parentification decrease and positive outcomes increase when family and sibling relationships are positive [36,71], or when a person presents proactive personality traits [63], and is able to view parentification experiences as fair [70].

## 7. Conclusions

The current study aimed to explore the relations between parentification and resiliency in the Polish adolescent sample. The study produced interesting results: (1) there were differences in resiliency and parentification dimensions between girls and boys, and (2) the relations between resiliency and parentification dimensions may be gender-specific within the Polish adolescent sample. The current findings can lay the foundation for future studies on the topic.

## Figures and Tables

**Table 1:** Summary Statistics, Reliability, and Correlations between Study Variables (*N* = 164).

Variable	*M*	*SD*	Min	Max	α	1	2	3	4	5	6	7	8	9	10	11
1. PCaNAT	2.377	0.931	0.00	4.55	0.818	–										
2. SoHaO	2.554	0.885	0.25	4.25	0.779	0.48 **	–									
3. PaD	2.679	0.778	0.60	4.15	0.825	0.55 **	0.32 **	–								
4. OAaE	2.535	0.797	0.25	4.55	0.799	0.70 **	0.51 **	0.62 **	–							
5. GR	2.514	0.746	0.05	4.15	0.910	0.73 **	0.62 **	0.68 **	0.726 **	–						
6. IPTP	1.857	0.758	1	4.45	0.715	0.08	0.12	0.22 **	0.174 *	0.18 *	–					
7. EPTP	2.960	0.926	1	5	0.723	0.04	−0.01	0.25 **	0.124	0.10	0.16 *	–				
8. SI	2.068	0.913	1	5	0.788	−0.15	−0.08	−0.15	−0.270 **	−0.11	0.12	0.06	–			
9. SWR	3.467	0.938	1	5	0.767	0.21 **	0.15	0.35 **	0.323**	0.27 **	0.13	0.23 **	−0.63 **	–		
10. IPTS	2.577	0.950	1	5	0.797	−0.01	0.02	0.28 **	0.048	0.14	0.16 *	0.46 **	−0.03	0.28 **	–	
11. EPTS	2.294	0.905	1	5	0.752	0.03	0.07	0.25 **	0.144	0.29 **	0.27 **	0.31 **	0.19 *	0.09	0.50 **	–

Note: PCaNAT = Personal Competencies and Negative Affect Tolerance; SoHaO = Sense of Humour and Openness to New Experiences; PaD = Persistence and Determination in Action; OAaE = Optimistic Attitude and Energy; GR = General Resiliency; IPTP = Instrumental Parentification toward Parents; EPTP = Emotional Parentification toward Parents; SI = Sense of Injustice; SWR = Satisfaction with the Role; IPTS = Instrumental Parentification toward Siblings; EPTS = Emotional Parentification toward Siblings; α = Cronbach’s alpha coefficient; * *p* < 0.05. ** *p* < 0.01.

**Table 2:** Gender Dfferences in Study Variables (*N* = 164).

Variable	Girls (*n* = 101)		Boys (*n* = 63)		*t*
	*M*	*SD*	*M*	*SD*	
Personal Competencies and Negative Affect Tolerance	2.19	0.94	2.68	0.85	−3.34 **
Sense of Humour and Openness to New Experiences	2.47	0.88	2.68	0.88	−1.47
Persistence and Determination in Action	2.57	0.76	2.85	0.79	−2.20 *
Optimistic Attitude and Energy	2.42	0.77	2.73	0.82	−2.47
General Resiliency	2.38	0.72	2.73	0.75	−2.97 *
Instrumental Parentification toward Parents	1.75	0.67	2.02	0.86	−2.20 *
Emotional Parentification toward Parents	3.07	0.97	2.78	0.82	2.01 *
Sense of Injustice	2.12	1.00	1.99	0.76	0.90
Satisfaction with the Role	3.35	1.00	3.66	0.80	−2.07 *
Instrumental Parentification toward Siblings	2.76	0.95	2.29	0.88	3.17 **
Emotional Parentification toward Siblings	2.31	0.89	2.26	0.94	0.36

Note: * *p* < 0.05. ** *p* < 0.01.

**Table 3:** R-Pearson Correaltions between Study Variables in the Subsample of Girls (*n* = 101).

Variable	PCaNAT	SoHaO	PaD	OAaE	GR
IPTP	−0.06	0.11	0.10	0.01	0.06
EPTP	0.06	−0.03	0.27 **	0.06	0.09
SI	−0.13	−0.09	−0.23 *	−0.39 **	−0.17
SWR	0.14	0.18	0.32 **	0.37 **	0.25 *
IPTS	0.04	0.07	0.31 **	0.06	0.20 *
EPTS	−0.04	0.08	0.21 *	0.01	0.21 *

Note: PCaNAT = Personal Competencies and Negative Affect Tolerance; SoHaO = Sense of Humour and Openness to New Experiences; PaD = Persistence and Determination in Action; OAaE = Optimistic Attitude and Energy; GR = General Resiliency; IPTP = Instrumental Parentification toward Parents; EPTP = Emotional Parentification toward Parents; SI = Sense of Injustice; SWR = Satisfaction with the Role; IPTS = Instrumental Parentification toward Siblings; EPTS = Emotional Parentification toward Siblings; * *p* < 0.05. ** *p* < 0.01.

**Table 4:** R-Pearson Correaltions between Study Variables in the Subsample of Boys (*n* = 63).

Variable	PCaNAT	SoHaO	PaD	OAaE	GR
IPTP	0.18	0.11	0.32 *	0.31 *	0.26 *
EPTP	0.13	0.08	0.32 *	0.33 **	0.23
SI	−0.13	−0.03	0.03	−0.03	0.05
SWR	0.28 *	0.03	0.34 **	0.18	0.25 *
IPTS	0.07	0.02	0.38 **	0.16	0.22
EPTS	0.17	0.07	0.33 **	0.36 **	0.45 **

Note: PCaNAT = Personal Competencies and Negative Affect Tolerance; SoHaO = Sense of Humour and Openness to New Experiences; PaD = Persistence and Determination in Action; OAaE = Optimistic Attitude and Energy; GR = General Resiliency; IPTP = Instrumental Parentification toward Parents; EPTP = Emotional Parentification toward Parents; SI = Sense of Injustice; SWR = Satisfaction with the Role; IPTS = Instrumental Parentification toward Siblings; EPTS = Emotional Parentification toward Siblings; * *p* < 0.05. ** *p* < 0.01.

## Data Availability

The data presented in this study are available on request from the corresponding author.
